# Central nervous system schwannoma, VGLL-fused (EWSR1::VGLL1 fusion) with neuroblastoma-like cell dense areas in the frontal lobe of a young man with schwannomatosis due to a germline LZTR1 mutation

**DOI:** 10.17879/freeneuropathology-2026-8898

**Published:** 2026-01-08

**Authors:** David G. Munoz, Sunit Das, Ju-Yoon Yoon, Robert Siddaway, Adrian Levine, Kenneth D. Aldape

**Affiliations:** 1 Laboratory Medicine and Pathobiology, University of Toronto, Toronto, ON, Canada and Department of Laboratory Medicine, Unity Health Toronto, Toronto, ON, Canada; 2 Department of Surgery, Division of Neurosurgery, Unity Health Toronto, Toronto, ON, Canada; 3 Department of Laboratory Medicine, Hospital for Sick Children, Toronto, ON, Canada; 4 Laboratory of Pathology, Center for Cancer Research, National Cancer Institute, National Institutes of Health, Bethesda, MD, USA

**Keywords:** EWSR1::VGLL1 fusion, LZTR1 mutation, Central nervous system schwannoma

## Abstract

We report a central nervous system schwannoma, *VGLL*-fused in a young man’s frontal lobe. Somatic abnormalities included an *EWSR1::VGLL1* fusion, which incorporated the entire translated region of *VGLL1*, but excluded most domains of *EWSR1*. The tumor histologically merged with the brain, and showed both schwannoma-like and neuroblastoma-like areas. A germline *LZTR1 *mutation was subsequently identified, implying the patient suffered from schwannomatosis.

A 29-year-old previously healthy man of Nepalese ethnicity suffered a tonic clonic seizure at home and was taken to the hospital, where a second seizure was witnessed. There was no family history of neurological disease and neurological exam was unremarkable.

Head magnetic resonance imaging (MRI) showed an avidly enhancing well-circumscribed intra-axial nodule in the left superior frontal gyrus extending to cortex, measuring 1.2 x 0.8 x 1.2 cm, and surrounded by vasogenic edema. It was hypointense on T2-weighted sequence. Diffusion weighted imaging showed no diffusion restriction. There was no blooming on susceptibility-weighted image. Computed tomography (CT) scan showed calcification. The main concern was solitary brain metastasis. (**Fig. 1**)

**Figure 1.**
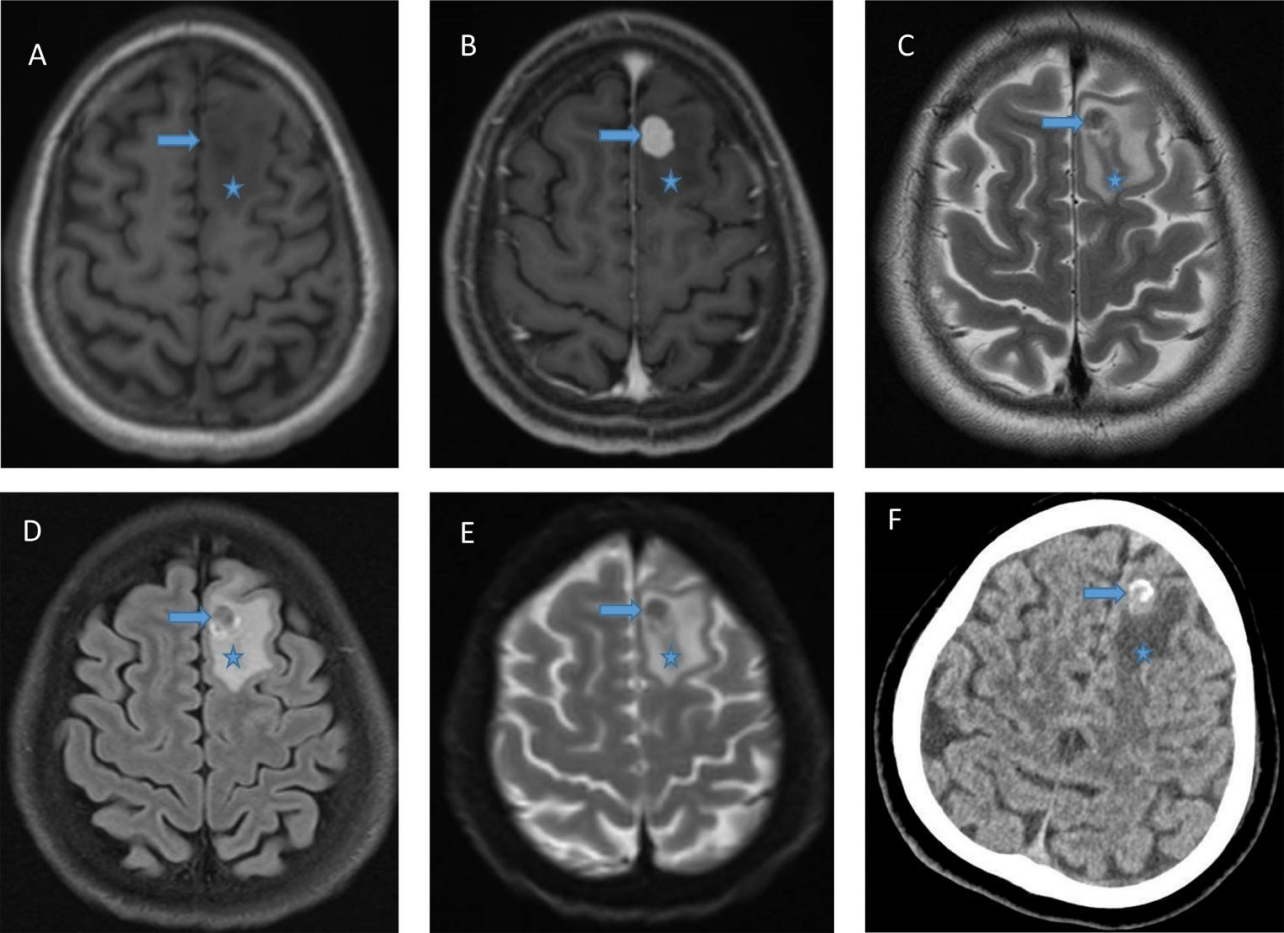
**A**. MRI: T1 showing circumscribed intra-axial nodule in the left superior frontal gyrus; **B**. MRI: T1 with gadolinium showing avid enhancing; **C**. MRI: T2 showing hypointensity of the lesion; **D**. MRI: FLAIR; **E**. MRI: Diffusion weighted imaging showing absence of diffusion restriction; **F**. CT scan with hyperdense areas construed as calcification by radiologists. In all images, arrow indicates tumor, and asterisk edema.

At surgery, the mass was intra-axial, and appeared firm and well demarcated from the brain. Using microsurgical technique, the tumor was mobilized from the adjacent white matter and resected en bloc. Post-surgical MRI confirmed complete resection. CT of the chest, abdomen and pelvis showed no evidence of systemic disease. No recurrence was identified after follow-up for 17 months.

A single firm white tan tissue mass measuring 1.0 x 0.9 x 0.6 cm was received in Pathology. Paraffin-embedded sections showed a biphasic tumor (**Fig. 2A**). The larger part was made up of cells with ovoid nuclei and cytoplasm of indistinct borders, arranged in streams organized in rhythmic palisades in a collagenous background (**Fig. 2B**), best demonstrated on Hematoxylin-Phloxine-Saffron (HPS) stain (**Fig. 2C**). The second component of the tumor consisted of an attached nodule of densely packed cells with round to ovoid nuclei containing a single prominent nucleolus, surrounded by scant cytoplasm lacking distinctive borders. The cells were often arranged around nuclei free areas containing fibrillary material, reminiscent of Homer-Wright rosettes (**Fig. 2D**). The primitive neuroepithelial appearance was considered neuroblastoma-like. Neither ganglion cells nor cells with marked cellular pleomorphism were identified. At the margins, the tumor nodules not only intermingled with the adjacent brain, but appeared to blend into it. Numerous Rosenthal fibers were present, predominantly at the periphery of the tumor, both in the neuropil and tumor tissue (**Fig. 2E**). Mitotic activity was minimal. There were no areas of necrosis. Reticulin stains demonstrated a dense network in both areas, consisting of both thin fibers investing individual cells, and thick fiber bundles (**Fig. 2F**).

**Figure 2.**
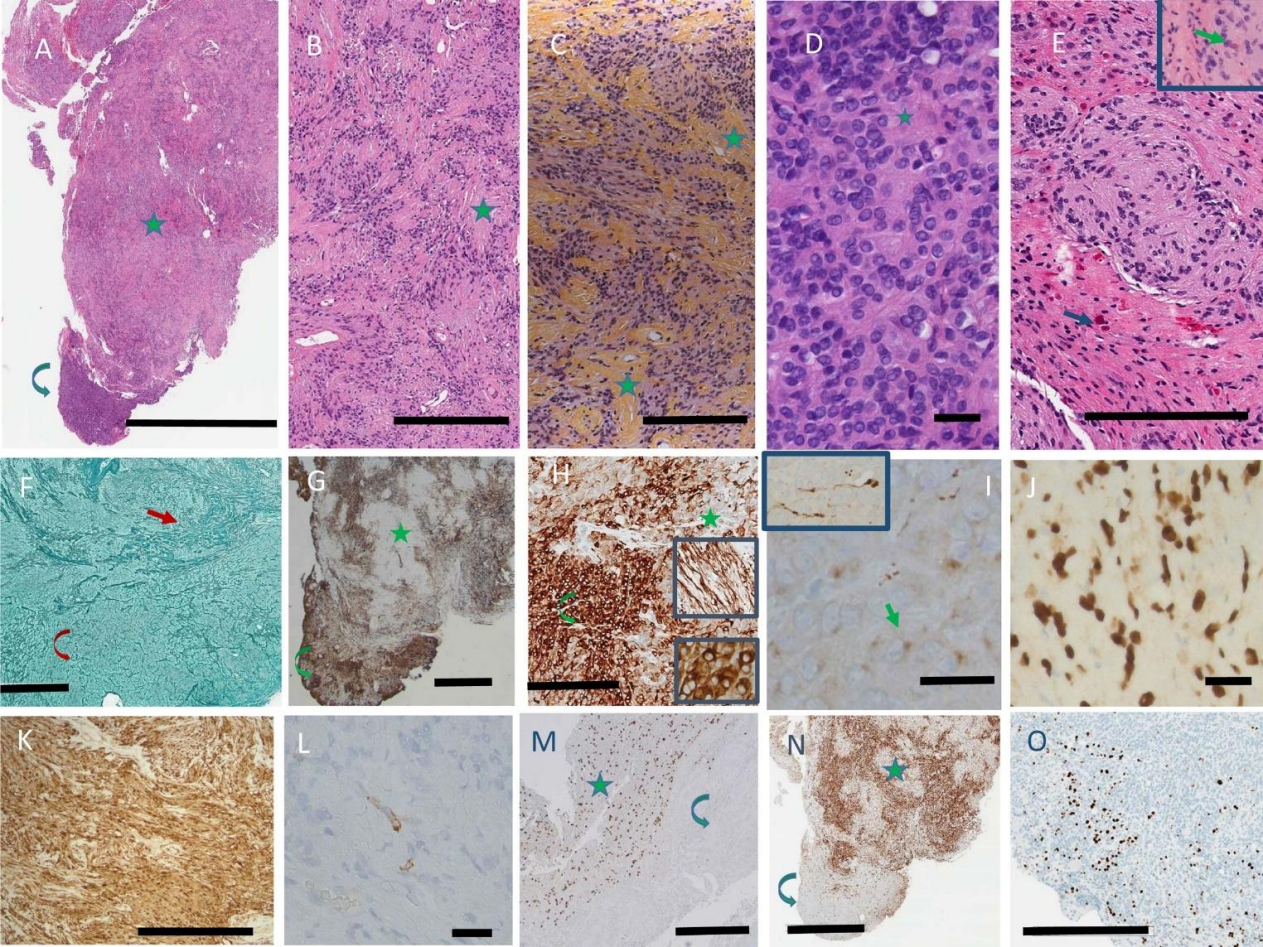
**A**. H&E, low power view, showing the edge with the neuropil, the bulk of the tumor’s schwannoma-like area with nuclear palisading in a collagenous background (star), and the neuroblastoma-like area (curved arrow). Bar: 3 mm. **B, C**. Schwannoma-like area, stained with H&E (**B**) and HPS (**C**) to highlight the rhythmic palisades in a collagenous background. Some examples are marked by stars. Bar: 300 μm. **D**. Neuroblastoma-like area with Homer-Wright rosettes (one marked by star). Bar: 25 μm. **E**. Rosenthal fibers (arrows), predominantly located in the intermingled neuropil, but a few within the tumor (inset), H&E. Bar: 200 μm. **F**. Reticulin stain, showing thick fibers in the schwannoma-like area (arrow) and thin fibers in the neuroblastoma-like area (curved arrow). Bar: 400 μm. **G**. GFAP, low power, highlighting the patchy distribution in both areas (same symbols as A). Bar: 1 mm. **H**. GFAP, high power showing a compact perinuclear pattern in the neuroblastoma-like areas (inset, lower panel), and processes in the schwannoma-like areas (inset, upper panel; same symbols as A). Bar: 200 μm. **I**. Synaptophysin, neuroblastoma-like area, with dot-like cytoplasmic label (arrow). Inset: axons with terminal swellings. Bar: 25 μm. **J**. SOX10. Bar: 25 μm. **K**. S100. Bar: 300 μm. **L**. EMA. Bar: 25 μm. **M**. OLIG2, showing labelling restricted to the adjacent neuropil (star), absent in the tumor (curved arrow). Bar: 400 μm. **N**. P16, predominantly expressed in the schwannoma-like area (same symbols as A). Bar: 2 mm. **O**. Ki-67. Bar 400 μm. Clicking here will lead you to the full H&E virtual slide.

Expression of GFAP was patchy in both areas: thin long processes were prominent extensions of the cytoplasm in the densely desmoplastic areas, whereas in the neuroblastoma-like areas GFAP was expressed in a compact perinuclear fashion (**Fig. 2G,H**). Virtually all tumor cells expressed SOX10 and S100 (**Fig. 2J,K**). Epithelial membrane antigen labeled a few scattered cells throughout the tumor (**Fig. 2L**). No axons were labeled by neurofilament stains. There was no expression of OLIG2 (**Fig. 2M**). ATRX was retained in tumor cell nuclei. Immunostaining with a monoclonal antibody specific for IDH1 R132H mutation showed no expression. Immunostaining for BRAF V600E was negative. In the neuroblastoma-like areas, synaptophysin labeled a small perinuclear cytoplasmic crescent, as well as several axons with terminal swellings (**Fig. 2I**). There was no synaptophysin expression in the desmoplastic areas, but at the edge with normal brain synaptophysin highlighted intermingling of tumor and normal neuropil. P16 intensely preferentially labeled the schwannoma-like component, with minimal labelling of the neuroblastoma-like areas of the tumor (**Fig. 2N**). CD34 was restricted to blood vessels. The Ki-67 proliferation index was estimated at 5 % in the desmoplastic component, but focally reached 10 % in the neuroblastoma-like component (**Fig. 2O**). Phosphohistone H3 showed rare mitotic figures in the desmoplastic component.

Extracted DNA from the paraffin block was subjected to comprehensive genomic profiling using Illumina TruSight PanCancer next generation sequencing (NGS), which revealed an *EWSR1::VGLL1* fusion, specifically *EWSR1* NM_013986 (exon 9, 331 AA) to *VGLL1 *NM_016267 (5’ UTR, exon 2) (**Fig. 3**). Although the *VGLL1* breakpoint was in the 5' UTR, the frame from *EWSR1* was maintained and met the ATG of *VGLL1* with insertion of the following amino acid sequence: CHLCHSLT. In addition, a tier II mutation in *LZTR1* (p.Trp265* (NM_006767.4:c.794G>A) was identified by hybridization-capture NGS assay. Following additional consent, a blood sample confirmed the presence of this mutation in the germline. The patient was referred for genetic counselling.

**Figure 3.**
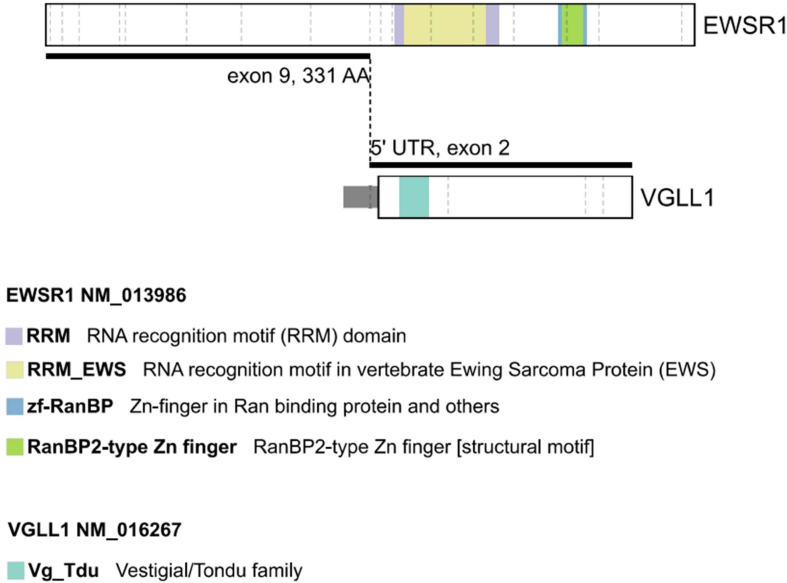
Diagram of the *EWSR1::VGLL1* fusion. Note that the *VGLL1* breakpoint was in the 5' UTR, and thus the entire reading frame was incorporated, including the Vestigial/Tondo family domain. In contrast, none of the labeled domains in *EWSR1* were included in the fused transcript.

A methylation profile analysis was carried out at the National Cancer Institute. The composite methylation profile, on versions 11b6 and 12b6 of the Heidelberg classifier, as well as the NCI/Bethesda classifier, indicated a consensus match to CNS Schwannoma, VGLL-fused. Dimensionality reduction with Uniform Manifold Approximation and Projection (UMAP) also placed this tumor in the same class. The analysis included both the schwannoma-like and neuroblastoma-like areas of the tumor without separating them. Single-nucleus RNA-sequencing was not performed.

## Discussion

This frontal lobe tumor, which histologically intermingled with the neuropil and contained a mixture of areas recognizable as schwannoma and others of primitive appearance reminiscent of neuroblastoma, was only correctly diagnosed following the demonstration of an* EWSR1::VGLL1* fusion. Tumors histologically recognizable as schwannomas but lacking any relationship to cranial nerves are considered intracerebral schwannomas. As many as 70 have been described since the first description in 1966 [1]. Some have been reported in association with neurofibromatosis type 2 [2], and recent reports describe loss of chromosome 22q. A novel category, recognized as a new tumor entity using DNA methylation-based brain tumor classifier is the vestigial-like family (*VGLL*)-altered central nervous system (CNS) schwannoma (VGLLACS). Schmid and others [3] have described the clinical, radiological, histological, and molecular features of 20 such tumors. An *EWSR1::VGLL1* fusion had been reported previously in "pediatric neuroepithelial neoplasm" [4] (probably a VGLLACS), and peripheral hybrid nerve sheath tumors combining features of schwannoma and perineuroma often harbor *VGLL3* fusions [5]. The latter do not group with VGLLACS in methylation profile dimensionally reduction analysis, however [3].

In Schmid et al. series [3], VGLLACS patients were aged 7 to 75 years of age, always lacking a family history. Supratentorial was the most common location. Areas with the histological pattern of schwannoma were combined in 54 % of cases with interspersed CNS tissue. This was reinforced by the presence of Rosenthal fibers not only in the interspersed or surrounding CNS tissue, but also within solid tumor in at least two cases. One of their 20 cases is reported to have neuroblastoma-like cell dense areas, not unlike our case. Thus, the histologic features of our case are consistent with an unusual, previously described morphology of VGLLACS. Likewise, the intense homogeneous enhancement in the absence of diffusion restriction are the imaging features seen in our case and considered characteristic by Schmid et al.. *VGLL1*-fused cases could be supratentorial, infratentorial, or even on occasions spinal whereas all *VGLL3*-fused cases were supratentorial. The fusion partner could be *EWSR1*, as in our patient, but even more frequently *CHD7*, and rarely *SS18*. Upregulation of *VGLL1*, but not of *EWSR1*, was identified in the only case where single nucleus RNA sequencing was performed [3]. This is consistent with *VGLL* acting as an oncogene (rather than a tumor suppressor). A conserved domain (TDU motif) preserved in the fusion moiety in our case, is considered critical for the *VGLL* binding to the transcription factor TEAD, a critical step in the Hippo pathway [6]. This fragment contains the homologous sequence to the vestigial (Vg) gene of Drosophila melanogaster. In humans, *VGLL1* is exclusively expressed in the placenta, a tissue distribution shared with frizzled homologue 10 (*FZD10*), an oncogene overexpressed in synovial sarcomas [7]. The involvement of *VGLL3* in neural crest development provides a rationale for altered migratory behavior of Schwann cells precursors [8]. The *EWSR1::VGLL1* fusion was first described in malignant myoepithelial tumor, a neoplasm of soft tissue [9]. In this soft tissue category, *VGLL3* along with other fusion partners is associated with inflammatory fibroblastic tumor and rhabdomyosarcoma [10].

In our case, as in Schmid et al., next generation sequencing did not find any of the alterations typically found in schwannomas (*NF2, LATS1, LATS2, ARIDA1, ARID1B, DDR1*, and specially *SOX10* in frame indels) [11]. Several cases of intracranial schwannoma with NF2 have been reported, but their *VGLL* fusion status is not known [12]. Recently Nguyen et al. have described a VGLLACS adjacent to the horn of the lateral ventricle in an infant with a germline TSC2 mutation [13]. Schmid et al. reported likely pathogenic variants in *EWSR1* and *SH2B3*, but no recurrent other mutations were identified in their VGLLACS series. However, in addition to the *EWSR1::VGLL1* fusion, our patient’s tumor had a nonsense mutation in *LZTR1*, one of the two genes with germ-line mutations implicated in non-NF2 associated schwannomatosis [14]. The mutation, present in the germ line as well as in the tumor, is predicted to result in a truncated gene product. A mutation in *LZTR1* has been described once in an intracranial schwannoma of unknown VGLL status [15]. *LZTR1* mutations are commonly encountered in glioblastoma [16], where point mutations are often associated with copy number losses. In addition, two families have been reported where one member had schwannomatosis and another member developed a glioblastoma containing the *LZTR1* mutation [17]. *LZTR1*, which stands for leucine zipper like transcription regulator 1, is an adapter of the cullin 3 protein, involved in ubiquitination of RAS. Mutations and deletions disrupt the function of *LZTR1* leading to activation of the RAS/MAPK signaling pathway [18].

Our case, as those in Schmid et al., has short follow-up, which limits the evaluation of the prognostic significance of the described genetic alterations. In conclusion, neuroblastoma-like areas are present in a small subset of VGLLACS, and our case is the second reported. We show that VGLLACS can be the presentation of schwannomatosis, in this case with a *LZTR1 *mutation*.*

## Conflict of interest statement

The authors declare no conflict of interest.
